# Time-to-Event Modeling for Survival Prediction of Osimertinib as the First- and Second-Line Therapy

**DOI:** 10.3390/jcm14124077

**Published:** 2025-06-09

**Authors:** Sungjae Lee, Heungjo Kim, Hongjae Lee, Jongsung Hahn, Min Jung Chang

**Affiliations:** 1 Department of Pharmacy and Yonsei Institute of Pharmaceutical Sciences, Yonsei University, Incheon 21983, Republic of Korea; chao1984@naver.com (S.L.); heungjo@yonsei.ac.kr (H.K.); manu5315@yonsei.ac.kr (H.L.); 2 Department of Pharmaceutical Medicine and Regulatory Science, Yonsei University, Incheon 21983, Republic of Korea; 3 Department of Pharmacy, Jeonbuk National University, Jeonju-si 54896, Republic of Korea; jongsung@jbnu.ac.kr; 4 Graduate Program of Industrial Pharmaceutical Science, Yonsei University, Incheon 21983, Republic of Korea

**Keywords:** non-small cell lung cancer (NSCLC), time-to-event modeling, Osimertinib

## Abstract

**Objectives:** To predict the survival rates of Osimertinib as first- and second-line therapy using time-to-event models based on literature data. **Methods:** Kaplan–Meier curves from randomized clinical trials were extracted after a systematic search of PubMed and Cochrane Library from their inception to 10 May 2023. Randomized clinical trials of Osimertinib reporting both first- and second-line overall survival (OS) and progression-free survival (PFS) in NSCLC patients with specific mutations, compared to earlier epidermal growth factor receptor (EGFR) inhibitors and chemotherapy. Kaplan–Meier curves of OS and PFS were extracted from published articles. A two-column raw dataset (time, survival probability) was extracted, and time-to-event outcomes (time, event) were derived using a graphic reconstructive algorithm. Data analysis was conducted from 1 June 2023 to 31 January 2024. Primary outcomes included OS and PFS for time-to-event modeling of Osimertinib as first- and second-line therapy. **Results:** The Weibull model, incorporating race as a covariate, best fit the first-line OS data. The log-logistic model best fit first-line PFS and second-line OS/PFS data. Based on these models, the predicted median OS for first-line and second-line treatment were 36.35 months (95% CI, 33.53–39.30 months) and 27.46 months (95% CI, 25.30–29.99 months), respectively. The predicted median PFS were 18.11 months (95% CI, 16.37–19.90 months) and 10.35 months (95% CI, 9.31–11.44 months), respectively. The predicted 3- and 5-year survival rates with first-line Osimertinib were 51% and 23%, respectively. Subgroup analysis revealed longer estimated 3- and 5-year survival rates for non-Asian patients compared to Asian patients (60% vs. 49% and 29% vs. 21%, respectively). **Conclusions:** The predicted survival rates from the time-to-event modeling align with the original clinical trial results, and an ethnic difference in Osimertinib efficacy was observed.

## 1. Introduction

Lung cancer remains a leading cause of cancer mortality worldwide, accounting for 1.4 million deaths annually, with a five-year survival rate of less than 20% [1,2]. Despite platinum-based combination chemotherapy, the overall survival (OS) for most patients with advanced non-small cell lung cancer (NSCLC) remains less than one year [3]. Recent years have seen a paradigm shift in the treatment of advanced NSCLC due to the identification of somatic sensitizing epidermal growth factor receptor (EGFR) gene mutations, such as exon 19 deletion (Ex19del) or exon 21 (L858R) substitution, as targets for tyrosine kinase inhibitors (TKIs). Owing to their superior efficacy compared to chemotherapy, first- and second-generation EGFR TKIs, including erlotinib, gefitinib, and afatinib, are now approved for first-line treatment of patients with advanced NSCLC harboring sensitizing EGFR mutations. Following the introduction of these EGFR TKIs, life expectancy in NSCLC patients with EGFR mutations increased from 21.6 to 28.2 months in OS [4,5,6] and from 9.5 to 13.1 months in progression-free survival (PFS) [4,7,8]. However, patients with advanced NSCLC treated with first- and second-generation EGFR TKIs inevitably develop acquired resistance, with the most common mechanism being a threonine-to-methionine substitution (T790M) secondary mutation at exon 20 in EGFR, occurring in 50–60% of treated patients [9,10]. This prompted the development of third-generation EGFR TKIs, such as Osimertinib, specifically designed to target T790M and EGFR TKI sensitizing mutations with greater selectivity over the wild-type form [11,12].

The outcomes of the FLAURA study underscore significant advancements in treating NSCLC patients with EGFR mutations using Osimertinib as a first-line treatment. This study demonstrated Osimertinib’s superiority over standard EGFR TKIs, such as gefitinib or erlotinib, by substantially extending progression-free survival (PFS) and OS rates. Specifically, Osimertinib extended median PFS to 18.9 months compared to 10.2 months with first-generation EGFR TKIs (gefitinib or erlotinib) and increased median OS to 38.6 months compared to 31.8 months with comparator EGFR TKIs [13,14]. Consequently, the United States Food and Drug Administration (FDA) has recently approved Osimertinib for the first-line treatment of patients with EGFRm (Ex19del or L858R) metastatic NSCLC [15]. Additionally, the National Comprehensive Cancer Network (NCCN) Clinical Practice Guidelines in Oncology for NSCLC [16] now recommend Osimertinib as the first-line treatment option for patients with EGFR activating mutation-positive NSCLC. However, evidence regarding the clinical efficacy of Osimertinib remains limited due to insufficient data from head-to-head trials across several phases and factors. Moreover, the FLAURA study indicated that the OS data were 58% mature at the time of the planned analysis. To enhance our understanding of Osimertinib’s long-term survival potential beyond the observed follow-up period from the FLAURA study, we conducted an analysis using a parametric survival model to estimate outcomes up to five years based on data from the FLAURA study.

Typically, the success of new anti-tumor drugs is assessed by their ability to improve OS and PFS in large, randomized, phase III trials, as OS has been demonstrated to be highly correlated with PFS. Evaluating the value of oncology drugs, particularly in terms of OS, requires data over an extended duration of several years. Comprehensive understanding is challenging because only one pivotal study is typically conducted due to time and cost constraints. Furthermore, no surrogate endpoints have yet proven to be reliable for OS and PFS in patients treated with cancer drugs. Time-to-event (TTE) modeling is a parametric analysis of the efficacy of anti-cancer drugs, which can be applied to predict the median time to achieve an outcome and to identify factors significantly affecting the outcome variable [17]. In this study, we developed a TTE model to predict the efficacy of Osimertinib as a first-line or second-line treatment and to identify factors influencing efficacy.

## 2. Materials and Methods

### 2.1. Literature Search

We systematically searched the PubMed and Cochrane Library electronic databases for articles published up to 10 May 2023. The search used the following keywords: ‘non-small cell lung cancer’ and ‘osimertinib’ and ‘first-line therapy’ and/or ‘second-line therapy’ and ‘progression-free survival’ and/or ‘overall survival’. The search was restricted to studies published in English and to clinical trials. Detailed information on the search strategies and inclusion/exclusion criteria is provided in Table S1 in the Supplementary Information.

### 2.2. Study Selection

We initiated the screening process by evaluating the articles based on their titles and abstracts. Subsequently, we reviewed the full text of each selected article to extract all the necessary information and verify whether the predefined inclusion criteria were met. We included eligible studies with OS and/or PFS data from clinical trials that compared the efficacy of Osimertinib to that of another TKI as first-line or second-line treatment in patients with NSCLC. We excluded studies with a sample size ≤50, abstract, subgroup or post hoc analyses, meta-analyses, summary reviews, retrospective research, and duplicated references. The study was not registered the systematic review registration information (e.g., PROSPERO). However, this study used the Preferred Reporting Items for Systematic Reviews and Meta-Analyses (PRISMA) reporting guidelines for Individual Patient Data (IPD) [18].

### 2.3. Data Extraction

For each study, the following information was extracted from the text, tables, and figures:

(1) Literature characteristics and study design (author, publication year, and sample size per arm), (2) demographic information (male–female ratio, age, race, Eastern Cooperative Oncology Group (ECOG) performance status), and (3) clinical outcomes (OS (time from randomization until death from any cause) and PFS (time from randomization until disease progression according to Response Evaluation Criteria in Solid Tumors (RECIST) or death from any cause)).

We extracted a two-column raw dataset (time and survival probability) from the Kaplan–Meier curves and final IPD datasets (time and event) of the points extracted from the raw dataset using IPDfromKM: Reconstruct IPD From Kaplan–Meier Survival Curve website (https://www.trialdesign.org/one-page-shell.html (accessed on 6 November 2023)) [19].

### 2.4. Modeling Analysis

This study used a parametric survival function to analyze the time courses of OS and PFS in patients with NSCLC treated with Osimertinib. In all analyses, the primary outcome was prespecified as OS and the secondary outcome was PFS. To select an appropriate basic model, three parametric TTE models (Weibull, Gompertz, and log-logistic) were developed using MonolixSuite (version 2023R1; Lixoft, Antony, France) and the stochastic approximation expectation maximization (SAEM) algorithm.

### 2.5. Model Development

Three hazard functions were evaluated: the Weibull (Equation (1)), Gompertz (Equation (2)), and log-logistic (Equation (3)) models [17,20,21].

(1) $$h\left(t\right)=\lambda \cdot \gamma \cdot t^{\gamma -1}$$

(2) $$h\left(t\right)=\lambda \cdot e^{\gamma \cdot t}$$

(3) $$h\left(t\right)=\frac{\lambda \cdot \gamma \cdot t^{\gamma -1}}{1+{\left(\lambda \cdot t\right)}^{\gamma }}$$

The hazard functions are defined using the parameters λ and γ, representing the risk rate at time 0 and the regression rate, respectively. The survival model is associated with the hazard function h(t), representing the instantaneous hazard at time t. Equation (4) describes the relationship between the survival and hazard functions.

(4) $$S\left(t\right)=\exp\left(-\int_{0}^{t}h\left(t\right)dt\right)$$

In Equation (4), $\int_{t}^{0}h\left(t\right)dt$ is the cumulative hazard from time 0 to t.

Three hazard functions were assessed to determine an appropriate structural model. The best model was selected based on the minimum value of the Akaike information criterion (AIC), relative standard errors (RSEs) of the model parameter estimates, and goodness-of-fit (GOF) plots [22].

Inter-study variability (Equation (5)) and residual variability (Equation (6)) were added to the structural model to account for the variation between the observed and model-predicted typical values:

(5) $$P_{i}=P_{typical}\times e^{\eta }$$

(6) $${Obs}_{i,j}={Pred}_{i,j}+\varepsilon_{i,j}{*SE}_{i,j}$$

(7) $${SE}_{i,j}=\sqrt{\frac{{Obs}_{i,j}*\left(1-{Obs}_{i,j}\right)}{N_{i,j}}}$$

In Equation (5), P_i_ is the hazard function parameter (λ or γ) of the individual study i; P_typical_ is the typical hazard function parameter of all studies; and η is the inter-study variation in the hazard function parameter, with a mean of 0 and a variance of *ω*^2^. In Equation (6), Obs_i,j_ is the observed probability of OS or PFS at the time point j of study i; Pred_i,j_ is the corresponding predicted value; and ε_i,j_ is a residual at the time point j of study i, which was assumed to be normally distributed with a mean of 0 and a variance of *σ*^2^. The residual error was weighted using the standard error of the corresponding probability of OS or PFS, which was calculated using Equation (7). In Equation (7), N_i_ represents the sample size at time point j in study i.

### 2.6. Covariate Model

Once the base model was established, the Wald test was used to screen for statistically significant covariates (*p* < 0.05) [23]. The covariates tested in this study included race, and the categorical covariates were introduced in Equation (8).

(8) $$P_{pop}=P_{typical}\times e^{\theta_{cov}\cdot COV}$$

In Equation (8), P_pop_ is the population prediction of the corresponding model parameter; P_typical_ represents the typical value of the model parameter; COV is the reported value of the tested covariate; and θ_cov_ is the scaling factor estimated effect of the covariate on the parameter.

### 2.7. Model Evaluation

After the final model was established, its performance was evaluated by visual inspection of the diagnostic GOF plots. A visual predictive check (VPC) was conducted by comparing the median and the 10th and 90th percentiles with the observed OS and PFS values to assess the prediction performance of the final model.

### 2.8. Simulation

Simulations and subgroup analyses were performed using Simulx (version 2023R1; Lixoft, Antony, France) to examine the progression of survival over time based on the final model. Our analytical method consists of two key steps. First, we obtain the model parameter estimates and their standard errors for each treatment group. Second, we pooled these estimated parameters within predefined subgroups using the scale and shape parameters of the final model to determine the median survival and 95% CI. Using these parameters, we derived the typical value for the time course in each subgroup through 1000 simulations per scenario using Simulx.

### 2.9. Risk of Bias and Quality Assessment 

We used a revised version (version 2.0; 22 August 2019) of the Cochrane Collaboration’s Risk of Bias tool [24] for quality assessment, according to the following categories: (1) bias arising from the randomization process, (2) bias due to deviations from intended interventions, (3) bias due to missing outcome data, (4) bias in the measurement of the outcome, and (5) bias in the selection of the reported result. We then determined whether each included trial was at low risk, had some concerns, or had a high risk of bias. The Kaplan–Meier curves of all IPD were extracted from randomized controlled trials (RCTs) to avoid any potential confounding factors. We constructed new survival curves from the reconstructed IPD and compared them with the original curves.

## 3. Results

### 3.1. Characteristics of the Included Studies

This study included four arms from six publications, with a sample size of 758 patients receiving Osimertinib as the first- and second-line therapy for NSCLC following the PRISMA guidelines (Tables S1 and S2, and Figure S1 in Supplementary Materials). Among these, two arms consisting of 350 participants were in the first-line treatment (FLAURA and FLAURA China), whereas two arms composed of 408 participants were in the second-line treatment (AURA2 and AURA3). Participants in the second-line treatment group had previously received first-line EGFR TKI treatment. All patients harbored sensitive EGFR mutations, specifically 19Del and L858R, and the tumors were locally advanced or metastatic. The treatment cycle was defined as 21 days of a once-daily treatment (80 mg/day). Five trials were conducted globally (in Caucasian, Asian, and other populations) and one trial was conducted in China. The AURA2 study used only second-line data and data from third-line or higher treatment options. Additionally, this study included the most recent OS results from the AURA2, AURA3, and FLAURA studies. A summary of the characteristics of the included studies is presented in Table 1.

### 3.2. Risk of Bias and Quality Assessment of Reconstructed Data

The risk of bias was determined to be low for all the studies, and the results are displayed in Figure S2 in the Supplementary Materials. The results indicated that the read-in dataset was a reliable representation of the original published KM curves, as shown in Figure S3 in the Supplementary Materials.

### 3.3. Overall Survival (OS)

The longitudinal OS probability curves based on the minimum AIC value were best described by the Weibull model for first-line therapy and the log-logistic model for second-line Osimertinib among the base models. The results of covariate screening indicated that race has a significant effect on the hazard rate of the Weibull model in first-line therapy, according to Wald statistics. Therefore, the effect of race on time to event (Te) was added to the final Weibull model as first-line therapy. As shown in Table S3 in Supplementary Materials, race was added to the Te parameters of the covariate model. However, there was no difference in the hazard function by race in second-line therapy because the same global patient population was used in AURA2 and AURA3. The TTE parameters of the study population are presented in Table 2.

**Figure 1:**
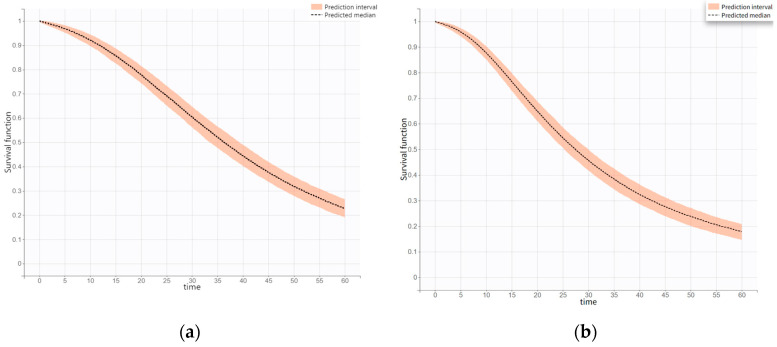
The simulation survival curves for final OS models as the first-line treatment (**a**) and second-line treatment (**b**), respectively.

The estimated median OS by simulation from final models in the first-line and second-line treatments were 36.35 months (95% CI, 33.53–39.30 months) and 27.46 months (95% CI, 25.30–29.99 months), respectively. The predicted 1-, 3- and 5-year survival rates with first-line Osimertinib were 90%, 51%, and 23%, respectively (Table S4). The simulated KM curves for the final OS models as first- and second-line treatments are shown in Figure 1. Based on the covariate modeling results, race was a statistically significant covariate for OS in first-line therapy. The groups were stratified according to the proportion of Asian and non-Asian participants for subgroup analysis. We simulated the same racial proportions as in the original study to identify racial differences accurately. The subgroup analysis in non-Asians had a significantly longer median OS (42.59 months, 95% CI, 39.16–45.58 months) than that in Asians (35.41 months, 95% CI, 32.76–38.39 months). It also showed a difference in 3- and 5-year survival rates for non-Asian patients compared to Asian patients (60% vs. 49% and 29% vs. 21%, respectively). Table S5 presents the results of the study.

The VPC of the final OS model as a diagnostic GOF is shown in Figure S4, which indicates that the final model fit well with the observed data.

### 3.4. Progression-Free Survival (PFS)

We chose the log-logistic model as the base model for both first- and second-line therapies. We also analyzed the influence of race only on first-line therapy because the same global patient population was used in AURA2 and AURA3 for second-line therapy. Wald statistics revealed that race in first-line therapy did not significantly affect the hazard rate of PFS. As shown in Table S6 in the Supplementary Materials, we selected the log-logistic model as the final model for both the first- and second-line therapies for PFS. The parameter estimates for the final model are presented in Table 3.

**Figure 2:**
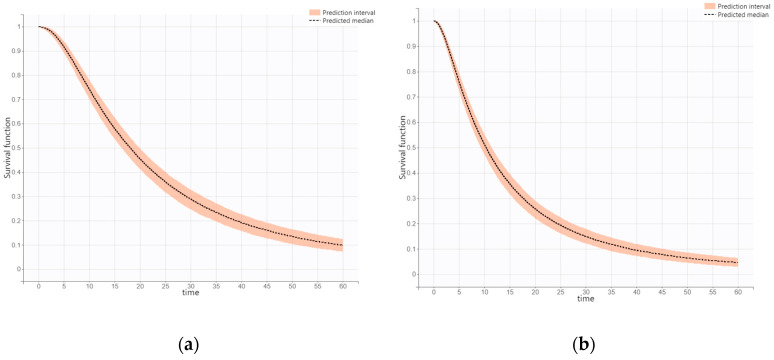
The simulation survival curves for final PFS models as the first-line treatment (**a**) and second-line treatment (**b**), respectively.

The estimated median PFS by simulation from final models for first-line and second-line therapies were 18.11 months (95% CI, 16.37–19.90 months) and 10.35 months (95% CI, 9.31–11.44 months), respectively. The simulated survival curves for the final PFS models as first- and second-line treatments are shown in Figure 2.

The VPC of the final PFS model, displayed as a diagnostic GOF plot, showed that the observed data generally fell within the range of the simulated percentiles (Figure S5).

## 4. Discussion

This study aimed to construct a survival model for Osimertinib as a first-line and second-line treatment in patients with NSCLC and to identify factors that impact its efficacy using TTE modeling based on pooled data extracted from clinical trials.

This approach allowed for the prediction of survival rates over a longer period than previously possible from the clinical trial data. The Weibull model, incorporating race as a covariate, demonstrated the optimal fit for overall survival data in the first-line treatment, whereas the log-logistic model best characterized PFS in the first-line treatment and both OS and PFS in the second-line treatment. Based on these final models, the predicted median OS for the first-line and second-line treatments were 36.35 months (95% CI, 33.53–39.30 months) and 27.46 months (95% CI, 25.30–29.99 months), respectively. Similarly, the predicted median PFS for the first-line and second-line treatments were 18.11 months (95% CI, 16.37–19.90 months) and 10.35 months (95% CI, 9.31–11.44 months), respectively. The observed OS and PFS data largely fell within the CI predicted by the final models, indicating good predictive accuracy. Furthermore, the estimated 3-year survival rate for the first-line treatment was 51%. The final model demonstrated strong concordance with the original data, accounting for 54% maturity (out of 58% overall maturity) in the FLAURA study and 39% (out of 65% overall maturity) in the FLAURA China study. To estimate the long-term survival potential of Osimertinib beyond the trial follow-up, TTE modeling with parametric survival models was used to project survival rates up to 5 years. The estimated 5-year survival rate from the final model in the first-line treatment was higher with Osimertinib than with gefitinib or erlotinib, as reported by Lin et al. (23% vs. 14.6%) [29].

In subgroup analysis, we found that ethnic differences, especially according to the proportion of Asian patients, significantly affected OS in patients receiving Osimertinib as first-line therapy. The simulation results showed that the median OS decreased with an increasing proportion of Asian patients, with 35.41 months and 42.59 months for Asians and non-Asians, respectively. Additionally, the estimated 3-year survival rate was higher for non-Asians than for Asians (60% vs. 49%), and the estimated 5-year survival rate was also higher for non-Asians than for Asians (29% vs. 21%). A critical insight from this study is that race is an essential factor affecting OS in patients who received Osimertinib treatment, and racial differences should be considered when conducting clinical trials.

In recent years, the medication Osimertinib, a third-generation EGFR TKI, has been extensively utilized as a first- and second-line treatment option for individuals diagnosed with advanced NSCLC harboring EGFR mutations. This widespread use is based on the superior OS and PFS data for Osimertinib compared to other EGFR TKIs, as reported in the FLAURA study. Despite the reimbursement approval for Osimertinib in nations such as Korea, China, and Japan [30], its effectiveness as a first-line treatment remains unclear due to the failure to improve OS in the Asian patient cohort and the L858R mutation patient groups [31]. A study conducted by Kim ES et al. reported that Osimertinib confers a significant overall survival benefit over erlotinib/gefitinib in non-Asian patients, but this benefit is not apparent in Asian patients [32]. Furthermore, another investigation by Chan et al. also revealed that in Asian patients harboring the L858R mutation, EGFR TKIs, including Osimertinib and combination treatments, showed no OS benefit compared to conventional chemotherapies [33]. Additionally, the ethnic differences in the population pharmacokinetics of Osimertinib are still a subject of controversy. While a study by Brown K et al. reported that the covariate model presented several effects of race/ethnicity on the apparent clearance of the metabolite of Osimertinib (AZ5104) [34]. Another study by Planchard D et al. found that the systemic exposure to Osimertinib was not significantly altered by race [35]. Recently, the literature regarding population pharmacokinetics of Osimertinib reports an apparent clearance for AZ5104 of approximately 31.3 L/h, which is more than twice that of Osimertinib (14.3 L/h). These findings have been consistently observed across multiple clinical trials and popPK analyses, and the variability related to patient factors such as race, weight, and albumin is not considered clinically meaningful [36]. Despite the controversy regarding the efficacy of Osimertinib by race, EGFR mutation, and population pharmacokinetics, our results of TTE simulation analysis were similar to those of previous studies concerning ethnic differences (Asian vs. non-Asian) and predicted long-term survival up to 5 years. However, analysis by EGFR mutation (exon 19 deletion vs. L858R) could not be simulated by TTE modeling because only the studies by Soria et al. for OS and Ahn et al. for PFS reported figures by mutation. These findings highlight the need for individualized treatment strategies that consider not only ethnicity but also the prevalence of EGFR mutation (exon 19 deletion vs. L858R), pharmacokinetic differences, including variations in drug metabolism enzymes (e.g., CYP3A4, CYP3A5), concomitant medications and comorbidities. Further studies, including prospective studies with comprehensive molecular and clinical data, are required to evaluate the mechanisms underlying these ethnic differences and can be translated into clinical practice for all patient groups.

To the best of our knowledge, this is the first study to develop a survival model for the first- and second-line treatments with the medication Osimertinib for individuals diagnosed with EGFR-mutated advanced NSCLC. This model possesses the capability to predict long-term survival outcomes and identify differences across various ethnic populations. Our modeling investigation aimed to assess the discrepancy between the OS and PFS values obtained from previous clinical trials and the actual clinical results. Consequently, we were the first researchers to present long-term survival rates and highlight the variations in patients with NSCLC across diverse ethnicities, particularly between Asian and non-Asian patients. The modeling outcomes demonstrated a notable similarity between the simulation results acquired through modeling and the actual clinical findings. This TTE modeling provides long-term survival rates such as OS and PFS, furnishing updated and compelling evidence regarding the efficacy of Osimertinib. Additionally, this study suggests that in future developments of anti-cancer pharmaceuticals, predicting the survival rates (OS/PFS) between novel drugs and existing medications solely based on clinically published trial outcomes could be feasible. This approach could be valuable, potentially saving substantial time and reducing costs in drug development processes.

Our study has some limitations. First, we only assessed study-level data for the TTE modeling due to the use of extracted data from clinical trial figures and actual clinical outcomes, rather than rely on original patient-level data. There is a potential risk of bias if the quality of the curves is suboptimal or information is missing. Also, the direct assessment of influencing factors was not possible since the distribution of the summary-level covariates was absent or narrow. For these reasons, it was difficult to perform detailed subgroup analyses and assess heterogeneity in treatment effects. Second, this study utilized OS and PFS data solely from randomized clinical trials, which limits the applicability to a wider patient population in real-world studies [37]. As a result, the findings may not fully reflect the efficacy of Osimertinib in clinical practice. Additionally, studies that did not report median OS figures, including those with short observation periods, were excluded from our analysis.

## 5. Conclusions

Based on this study, we predicted long-term survival rates using TTE modeling. Moreover, significant differences in the OS were observed between Asian and non-Asian patients. Through our study, patients can achieve better outcomes through modeling and simulations, and the cost and time required to conduct clinical trials can be reduced.

## Figures and Tables

**Table 1:** Summary of characteristics.

Treatment	First-Line			Second-Line		
Study	FLAURA	FLAURA Updated OS	FLAURA China	AURA2 + AURA Extension	AURA3	AURA3 Updated OS
Author	J.-C. Soria et al. [13]	S.S. Ramalingam et al. [14]	Ying Cheng et al. [25]	MJ Ahn et al. [26]	T.S. Mok et al. [27]	VA Papadimitrakopoulou et al. [28]
Publication year	2018	2020	2021	2019	2017	2020
Race (n, (%))	White 101 (36) Asian 174 (62) Other 4 (1)		Asian 71 (100) Non-Asian 0 (0)	White 101 (36) Asian 174 (62) Black or African American 4 (1) Other 7 (2) Not reported 4 (1)	White 89 (32) Asian 182 (65) Other 8 (3)	
Sample size per arm (n)	279		71	129	279	
Male (%)	36		39	32	38	
Age (year)	64		60	63	62	
ECOG PS 0/1 (%)	40/60		10/90	37/63	NA	
OS (mon.)	38.6		33.1	26.5	26.8	
PFS (mon.)	18.9		17.8	9.7	10.1	

**Table 2:** Parameter estimation of base and final model results in OS.

**First-Line**				
**Parameter**	**Base Model**		**Covariate Model** **(Final Model)**	
	**Estimate**	**RSE (%)**	**Estimate**	**RSE (%)**
Fixed effect				
Te_pop	44.31 (95% CI, 39.97–48.42)	5.10	41.69 (95% CI, 38.16–46.62)	5.07
p_pop	2.56 (95% CI, 2.06–3.5)	13.6	2.25 (95% CI, 1.77–3.55)	10.4
beta_Te_Race_global	NA	NA	0.19 (95% CI, 0.016–0.39)	50.0
Standard Deviation of the Random Effects				
omega_Te	0.58 (95% CI, 0.35–0.85)	24.1	0.44 (95% CI, 0.22–0.98)	22.9
omega_p	0.54 (95% CI, 0.39–0.82)	25.4	0.54 (95% CI, 0.42–0.75)	15.1
**Second-Line**				
**Parameter**	**Base Model**		**Covariate Model** **(Final Model)**	
	**Estimate**	**RSE (%)**	**Estimate**	**RSE (%)**
Fixed Effect				
Te_pop	27.47 (95% CI, 25.43–30.57)	4.58	NA	NA
p_pop	3.5 (95% CI, 2.71–4.67)	13.2	NA	NA
beta_Te_Race_global	NA	NA	NA	NA
Standard Deviation of the Random Effects				
omega_Te	0.6 (95% CI, 0.53–0.75)	9.42	NA	NA
omega_p	0.75 (95% CI, 0.57–1)	14.2	NA	NA

CI—confidence interval; RSE—relative standard error; Te_pop—time to event in the population; P_pop—shape parameter in the population; ΩTe—standard deviation of the random effects of time to event.

**Table 3:** Parameter estimation of base and final model results in PFS.

	First-Line		Second-Line	
Parameter	Final Model		Final Model	
	Estimate	RSE (%)	Estimate	RSE (%)
Fixed Effect				
Te_pop	18.03 (95% CI, 15.97–19.94)	5.83	10.31 (95% CI, 9.25–11.71)	5.89
p_pop	5.88 (95% CI, 4.67–9.23)	18.8	5.92 (95% CI, 3.76–7.62)	21.9
beta_Te_Race_global	NA	NA	NA	NA
Standard Deviation of the Random Effects				
omega_Te	0.8 (95% CI, 0.68–0.93)	8.69	0.93 (95% CI, 0.84–1.08)	6.85
omega_p	0.81 (95% CI, 0.58–1.1)	19.3	0.74 (95% CI, 0.38–0.99)	16.2

CI—confidence interval; RSE—relative standard error; Te_pop—time to event in the population; P_pop—shape parameter in the population; ΩTe—standard deviation of the random effects of time to event.

## Data Availability

The data supporting the findings of this study are available from the corresponding author upon reasonable request.

## Supplementary Materials

The following supporting information can be downloaded at: https://www.mdpi.com/article/10.3390/jcm14124077/s1, Table S1. Search Information; Table S2. List of included studies; Table S3. Comparison of Akaike information criterion (AIC) between the base and covariate models in OS; Table S4. The predicted 12, 24, 36, 48, and 60 months survival rates with first-line Osimertinib; Table S5. Comparison of subgroup analysis according to the proportion of Asians and non-Asians for the final OS model in the first-line treatment; Table S6. Comparison of Akaike information criteria (AIC) between the base and covariate models in PFS; Figure S1. PRISMA diagram; Figure S2. Risk-of-bias assessment; Figure S3. Comparison of the original and reconstructed Curves in OS and PFS; Figure S4. Visual predictive check for final OS models between first- and second-line treatments; Figure S5. Visual predictive check for final PFS models between first- and second-line treatments.

## References

[1] Torre L.A., Bray F., Siegel R.L., Ferlay J., Lortet-Tieulent J., Jemal A. (2015). Global cancer statistics, 2012. CA Cancer J. Clin..

[2] Sung H., Ferlay J., Siegel R.L., Laversanne M., Soerjomataram I., Jemal A., Bray F. (2021). Global cancer statistics 2020: GLOBOCAN estimates of incidence and mortality worldwide for 36 cancers in 185 countries. CA Cancer J. Clin..

[3] Schiller J.H., Harrington D., Belani C.P., Langer C., Sandler A., Krook J., Zhu J., Johnson D.H. (2002). Comparison of four chemotherapy regimens for advanced non–small-cell lung cancer. N. Engl. J. Med..

[4] Zhou C., Wu Y., Chen G., Feng J., Liu X.-Q., Wang C., Zhang S., Wang J., Zhou S., Ren S. (2015). Final overall survival results from a randomised, phase III study of erlotinib versus chemotherapy as first-line treatment of EGFR mutation-positive advanced non-small-cell lung cancer (OPTIMAL, CTONG-0802). Ann. Oncol..

[5] Fukuoka M., Wu Y.-L., Thongprasert S., Sunpaweravong P., Leong S.-S., Sriuranpong V., Chao T.-Y., Nakagawa K., Chu D.-T., Saijo N. (2011). Biomarker analyses and final overall survival results from a phase III, randomized, open-label, first-line study of gefitinib versus carboplatin/paclitaxel in clinically selected patients with advanced non–small-cell lung cancer in Asia (IPASS). J. Clin. Oncol..

[6] Yang J.C.-H., Wu Y.-L., Schuler M., Sebastian M., Popat S., Yamamoto N., Zhou C., Hu C.-P., O’Byrne K., Feng J. (2015). Afatinib versus cisplatin-based chemotherapy for EGFR mutation-positive lung adenocarcinoma (LUX-Lung 3 and LUX-Lung 6): Analysis of overall survival data from two randomised, phase 3 trials. Lancet Oncol..

[7] Mok T.S., Wu Y.-L., Thongprasert S., Yang C.-H., Chu D.-T., Saijo N., Sunpaweravong P., Han B., Margono B., Ichinose Y. (2009). Gefitinib or carboplatin–paclitaxel in pulmonary adenocarcinoma. N. Engl. J. Med..

[8] Sequist L.V., Yang J.C.-H., Yamamoto N., O’Byrne K., Hirsh V., Mok T., Geater S.L., Orlov S., Tsai C.-M., Boyer M. (2013). Phase III study of afatinib or cisplatin plus pemetrexed in patients with metastatic lung adenocarcinoma with EGFR mutations. J. Clin. Oncol..

[9] Pao W., Chmielecki J. (2010). Rational, biologically based treatment of EGFR-mutant non-small-cell lung cancer. Nat. Rev. Cancer.

[10] Gazdar A. (2009). Activating and resistance mutations of EGFR in non-small-cell lung cancer: Role in clinical response to EGFR tyrosine kinase inhibitors. Oncogene.

[11] Juchum M., Günther M., Laufer S.A. (2015). Fighting cancer drug resistance: Opportunities and challenges for mutation-specific EGFR inhibitors. Drug Resist. Updates.

[12] Engel J., Becker C., Lategahn J., Keul M., Ketzer J., Mühlenberg T., Kollipara L., Schultz-Fademrecht C., Zahedi R.P., Bauer S. (2016). Insight into the inhibition of drug-resistant mutants of the receptor tyrosine kinase EGFR. Angew. Chem. Int. Ed..

[13] Soria J.-C., Ohe Y., Vansteenkiste J., Reungwetwattana T., Chewaskulyong B., Lee K.H., Dechaphunkul A., Imamura F., Nogami N., Kurata T. (2018). Osimertinib in untreated EGFR-mutated advanced non–small-cell lung cancer. N. Engl. J. Med..

[14] Ramalingam S.S., Vansteenkiste J., Planchard D., Cho B.C., Gray J.E., Ohe Y., Zhou C., Reungwetwattana T., Cheng Y., Chewaskulyong B. (2020). Overall survival with osimertinib in untreated, EGFR-mutated advanced NSCLC. N. Engl. J. Med..

[15] U.S. Food and Drug Administration (2018). TAGRISSO (Osimertinib) Highlights of Prescribing Information.

[16] Ettinger D.S., Wood D.E., Aisner D.L., Akerley W., Bauman J., Chirieac L.R., D’Amico T.A., DeCamp M.M., Dilling T.J., Dobelbower M. (2017). Non–small cell lung cancer, version 5.2017, NCCN clinical practice guidelines in oncology. J. Natl. Compr. Cancer Netw..

[17] Holford N. (2013). A time to event tutorial for pharmacometricians. CPT Pharmacomet. Syst. Pharmacol..

[18] Stewart L.A., Clarke M., Rovers M., Riley R.D., Simmonds M., Stewart G., Tierney J.F. (2015). Preferred reporting items for a systematic review and meta-analysis of individual participant data: The PRISMA-IPD statement. JAMA.

[19] Liu N., Zhou Y., Lee J.J. (2021). IPDfromKM: Reconstruct individual patient data from published Kaplan-Meier survival curves. BMC Med. Res. Methodol..

[20] Van Wijk R.C., Simonsson U.S. (2022). Finding the right hazard function for time-to-event modeling: A tutorial and Shiny application. CPT Pharmacomet. Syst. Pharmacol..

[21] Gao L., Chen R., Li T., Li L., Zheng Q. (2021). Quantitative Analysis of the Efficacy of PARP Inhibitors as Maintenance Therapy in Recurrent Ovarian Cancer. Front. Pharmacol..

[22] Akaike H. (1998). Information theory and an extension of the maximum likelihood principle. Selected Papers of Hirotugu Akaike.

[23] Lindbom L., Ribbing J., Jonsson E.N. (2004). Perl-speaks-NONMEM (PsN)—A Perl module for NONMEM related programming. Comput. Methods Programs Biomed..

[24] Sterne J.A., Savović J., Page M.J., Elbers R.G., Blencowe N.S., Boutron I., Cates C.J., Cheng H.-Y., Corbett M.S., Eldridge S.M. (2019). RoB 2: A revised tool for assessing risk of bias in randomised trials. BMJ.

[25] Cheng Y., He Y., Li W., Zhang H.-l., Zhou Q., Wang B., Liu C., Walding A., Saggese M., Huang X. (2021). Osimertinib versus comparator EGFR TKI as first-line treatment for EGFR-mutated advanced NSCLC: FLAURA China, a randomized study. Target. Oncol..

[26] Ahn M.J., Tsai C.M., Shepherd F.A., Bazhenova L., Sequist L.V., Hida T., Yang J.C., Ramalingam S.S., Mitsudomi T., Jänne P.A. (2019). Osimertinib in patients with T790M mutation-positive, advanced non–small cell lung cancer: Long-term follow-up from a pooled analysis of 2 phase 2 studies. Cancer.

[27] Mok T.S., Wu Y.-L., Ahn M.-J., Garassino M.C., Kim H.R., Ramalingam S.S., Shepherd F.A., He Y., Akamatsu H., Theelen W.S. (2017). Osimertinib or platinum–pemetrexed in EGFR T790M–positive lung cancer. N. Engl. J. Med..

[28] Papadimitrakopoulou V., Mok T., Han J.-Y., Ahn M.-J., Delmonte A., Ramalingam S., Kim S., Shepherd F., Laskin J., He Y. (2020). Osimertinib versus platinum–pemetrexed for patients with EGFR T790M advanced NSCLC and progression on a prior EGFR-tyrosine kinase inhibitor: AURA3 overall survival analysis. Ann. Oncol..

[29] Lin J.J., Cardarella S., Lydon C.A., Dahlberg S.E., Jackman D.M., Jänne P.A., Johnson B.E. (2016). Five-year survival in EGFR-mutant metastatic lung adenocarcinoma treated with EGFR-TKIs. J. Thorac. Oncol..

[30] Wu Y.-L., Planchard D., Lu S., Sun H., Yamamoto N., Kim D.-W., Tan D., Yang J.-H., Azrif M., Mitsudomi T. (2019). Pan-Asian adapted Clinical Practice Guidelines for the management of patients with metastatic non-small-cell lung cancer: A CSCO–ESMO initiative endorsed by JSMO, KSMO, MOS, SSO and TOS. Ann. Oncol..

[31] Cho B.C., Chewaskulyong B., Lee K.H., Dechaphunkul A., Sriuranpong V., Imamura F., Nogami N., Kurata T., Okamoto I., Zhou C. (2019). Osimertinib versus standard of care EGFR TKI as first-line treatment in patients with EGFRm advanced NSCLC: FLAURA Asian subset. J. Thorac. Oncol..

[32] Kim E.S., Melosky B., Park K., Yamamoto N., Yang J.C. (2021). EGFR tyrosine kinase inhibitors for EGFR mutation-positive non-small-cell lung cancer: Outcomes in Asian populations. Future Oncol..

[33] Chan S.-K., Choi H.C.-W., Lee V.H.-F. (2022). Overall survival benefits of first-line treatments for Asian patients with advanced EGFR-mutated NSCLC harboring L858R mutation: A systematic review and network meta-analysis. JTO Clin. Res. Rep..

[34] Brown K., Comisar C., Witjes H., Maringwa J., de Greef R., Vishwanathan K., Cantarini M., Cox E. (2017). Population pharmacokinetics and exposure-response of osimertinib in patients with non-small cell lung cancer. Br. J. Clin. Pharmacol..

[35] Planchard D., Brown K.H., Kim D.-W., Kim S.-W., Ohe Y., Felip E., Leese P., Cantarini M., Vishwanathan K., Jänne P.A. (2016). Osimertinib Western and Asian clinical pharmacokinetics in patients and healthy volunteers: Implications for formulation, dose, and dosing frequency in pivotal clinical studies. Cancer Chemother. Pharmacol..

[36] Johnson M., Lin Y.W., Schmidt H., Sunnaker M., Van Maanen E., Huang X., Rukazenkov Y., Tomkinson H., Vishwanathan K. (2025). Population Pharmacokinetics of Osimertinib in Patients With Non-Small Cell Lung Cancer. Pharmacol. Res. Perspect..

[37] Mauro M.J., Davis C., Zyczynski T., Khoury H.J. (2015). The role of observational studies in optimizing the clinical management of chronic myeloid leukemia. Ther. Adv. Hematol..

