# Supplementary material for: Time-to-Event Modeling for Survival Prediction of Osimertinib as the First- and Second-Line Therapy
Source: J Clin Med. 2025 Jun 9;14(12):4077. doi: 10.3390/jcm14124077 (PMC12194035; doi:10.3390/jcm14124077)
Supplement: Supplementary file 1 [file jcm-14-04077-s001.zip › jcm-3674915-supplementary.pdf]

Supplementary Online Content

Table S1. Search Information
Table S2. List of included studies
Table S3. Comparison of Akaike information criterion (AIC) between the base and covariate models in OS
Table S4. The predicted 12, 24, 36, 48, and 60 months survival rates with first-line Osimertinib
Table S5. Comparison of subgroup analysis according to the proportion of Asians and non-Asians for the final OS model in the first-line treatment
Table S6. Comparison of Akaike information criteria (AIC) between the base and covariate models in PFS

Figure S1. PRISMA Diagram
Figure S2. Risk-of-Bias Assessment
Figure S3. Comparison of the original and reconstructed Curves in OS and PFS
Figure S4. Visual predictive check for final OS models between first- and second-line treatments
Figure S5. Visual predictive check of the final PFS models between first- and second-line treatments

Authors have provided the following supplementary material to readers to highlight additional information about their work.

Table S1. Search Information

Pubmed

No.	Query	Results	Date
#1	Search: (Non-Small-Cell Lung cancer[MeSH Terms]) OR (Non-Small-Cell Lung cancer[Title/Abstract]) Filters: Clinical Trial	8446	10-May-2023
#2	Search: osimertinib Filters: Clinical Trial	152	10-May-2023
#3	Search: (((first line therapy) OR (first line treatment)) OR (second line therapy)) OR (second line treatment) Filters: Clinical Trial	16103	10-May-2023
#4	Search: (progression free survival) OR (overall survival) Filters: Clinical Trial	123151	10-May-2023
#5	#1 AND #2 AND #3 AND #4	44	10-May-2023
#6	Search English [Language] Filters: Clinical Trial	44	10-May-2023

Cochran Library

No.	Query	Results	Date
#1	MeSH descriptor: [Carcinoma, Non-Small-Cell Lung] explode all trees	16145	10-May-2023
#2	osimertinib	439	10-May-2023
#3	(((first line therapy) OR (first line treatment)) OR (second line therapy)) OR (second line treatment)	35400	10-May-2023
#4	(progression free survival) OR (overall survival)	69934	10-May-2023
#5	(#1 AND #2 AND #3 AND #4)	136	10-May-2023
#6	Search English [Language] Filters: Clinical Trial	133	10-May-2023

Table S2. List of included studies

First-line therapy

1. Soria JC, Ohe Y, Vansteenkiste J, Reungwetwattana T, Chewaskulyong B, Lee KH, Dechaphunkul A, Imamura F, Nogami N, Kurata T, Okamoto I, Zhou C, Cho BC, Cheng Y, Cho EK, Voon PJ, Planchard D, Su WC, Gray JE, Lee SM, Hodge R, Marotti M, Rukazenkov Y, Ramalingam SS; FLAURA Investigators. Osimertinib for untreated EGFR-mutated advanced non-small cell lung cancer. N Engl J Med. 2018 Jan 11;378(2):113-125. doi: 10.1056/NEJMoa1713137. Epub 2017 Nov 18. PMID: 29151359.
2. Ramalingam SS, Vansteenkiste J, Planchard D, Cho BC, Gray JE, Ohe Y, Zhou C, Reungwetwattana T, Cheng Y, Chewaskulyong B, Shah R, Cobo M, Lee KH, Cheema P, Tiseo M, John T, Lin MC, Imamura F, Kurata T, Todd A, Hodge R, Saggese M, Rukazenkov Y, Soria JC; FLAURA Investigators. Overall Survival with osimertinib treatment in untreated EGFR-mutated advanced NSCLC. N Engl J Med. 2020 Jan 2;382(1):41-50. doi: 10.1056/NEJMoa1913662. Epub 2019 Nov 21. PMID: 31751012.
3. Cheng Y, He Y, Li W, Zhang HL, Zhou Q, Wang B, Liu C, Walding A, Saggese M, Huang X, Fan M, Wang J, Ramalingam SS. Osimertinib Versus Comparator EGFR TKI as First-Line Treatment for EGFR-Mutated Advanced NSCLC: FLAURA China, A Randomized Study. Target Oncol. 2021 Mar;16(2):165-176. doi: 10.1007/s11523-021-00794-6. Epub 2021 Feb 5. PMID: 33544337; PMCID: PMC7935816.

Second-line therapy

4. Ahn MJ, Tsai CM, Shepherd FA, Bazhenova L, Sequist LV, Hida T, Yang JCH, Ramalingam SS, Mitsudomi T, Jänne PA, Mann H, Cantarini M, Goss G. Osimertinib in patients with T790M mutation-positive, advanced non-small cell lung cancer: Long-term follow-up from a pooled analysis of 2 phase 2

studies. Cancer. 2019 Mar 15;125(6):892-901. doi: 10.1002/cncr.31891. Epub 2018 Dec 4. PMID: 30512189.

5. Mok TS, Wu Y-L, Ahn M-J, Garassino MC, Kim HR, Ramalingam SS, Shepherd FA, He Y, Akamatsu H, Theelen WS, Lee CK, Sebastian M, Templeton A, Mann H, Marotti M, Ghiorghiu S, Papadimitrakopoulou VA; AURA3 Investigators. Osimertinib or Platinum-Pemetrexed in EGFR T790M-Positive Lung Cancer. N Engl J Med. 2017 Feb 16;376(7):629-640. doi: 10.1056/NEJMoa1612674. Epub 2016 Dec 6. PMID: 27959700; PMCID: PMC6762027.
6. Papadimitrakopoulou VA, Mok TS, Han JY, Ahn MJ, Delmonte A, Ramalingam SS, Kim SW, Shepherd FA, Laskin J, He Y, Akamatsu H, Theelen WSME, Su WC, John T, Sebastian M, Mann H, Miranda M, Laus G, Rukazenkov Y, Wu YL. Osimertinib versus platinum-pemetrexed for patients with EGFR T790M advanced NSCLC and progression on a prior EGFR tyrosine kinase inhibitor: AURA3 overall survival analysis. Ann Oncol. 2020 Nov;31(11):1536-1544. doi: 10.1016/j.annonc.2020.08.2100. Epub 2020 Aug 27. PMID: 32861806.

Table S3. Comparison of akaike information criterion (AIC) between the base and covariate models in OS. AIC of the final model in first-line osimertinib (A) and AIC of the final model in second-line (B), respectively.

(A) Comparison of AIC among models in the first-line

Model		AIC	p-value from wald
Base model	Weibull	1937.9	-
	Gompertz	1938.67	
	Log-logistic	1940.42	
Covariate model (Weibull model + race)	+ Race	1936.59	Significant ($4.57e^{-2}$)

(B) Comparison of AIC among models in the second-line

Model		AIC	p-value from wald
Base model	Weibull	2442.67	-
	Gompertz	2442.55	
	Log-logistic	2441.58	
Covariate model (Weibull model + race)	+ Race[a]	NA	-

Notes: [a] same race(global) in AURA2 and AURA3

Table S4. The predicted 12, 24, 36, 48, and 60 months survival rates with first-line Osimertinib

Treatment	Probabilities of survival (%)				
	12 months	24 months	36 months	48 months	60 months
First-line Osimertinib	90	71	51	34	23

Table S5. Comparison of subgroup analysis according to the proportion of Asian and Non-Asian for final OS model in first-line treatment

Subgroup	Median Survival (months)	95% CI (Low)	95% CI (High)	Difference	p-value
**group 1 (Asian 0%/non-Asian 100%)**	42.59	39.16	45.58	ref	ref
**group 2 (Asian 62%/non-Asian 38%)**	37.41	33.94	41.5	-5.18	not significant ($8.01e^{-2}$)
**group 3 (Asian 100%/non-Asian 0%)**	35.41	32.76	38.39	-7.18	**Significant ($1.65e^{-2}$)**

Table S6. Comparison of akaike information criterion (AIC) between the base and covariate models in PFS. AIC of the final model in first-line (A) and AIC of the final model in second-line (B), respectively.

(A) Comparison of AIC among models in the first-line osimertinib survival model

Model		AIC	p-value from wald
**Base model**	**Weibull**	1457.95	-
	**Gompertz**	1458.95	
	**Log-logistic**	**1457.59**	
**Covariate model (Log-logistic model + race)**	**+ Race**	1458.29	not significant ($3.14e^{-1}$)

(B) Comparison of AIC among models in the second-line osimertinib survival model

Model		AIC	p-value from wald
**Base model**	**Weibull**	1638.07	-
	**Gompertz**	1642.98	
	**Log-logistic**	**1637.37**	
**Covariate model (Log-logistic model + race)**	**+ Race**[a]	NA	-

Notes: [a] same race(global) in AURA2 and AURA3

Figure S1. PRISMA Diagram

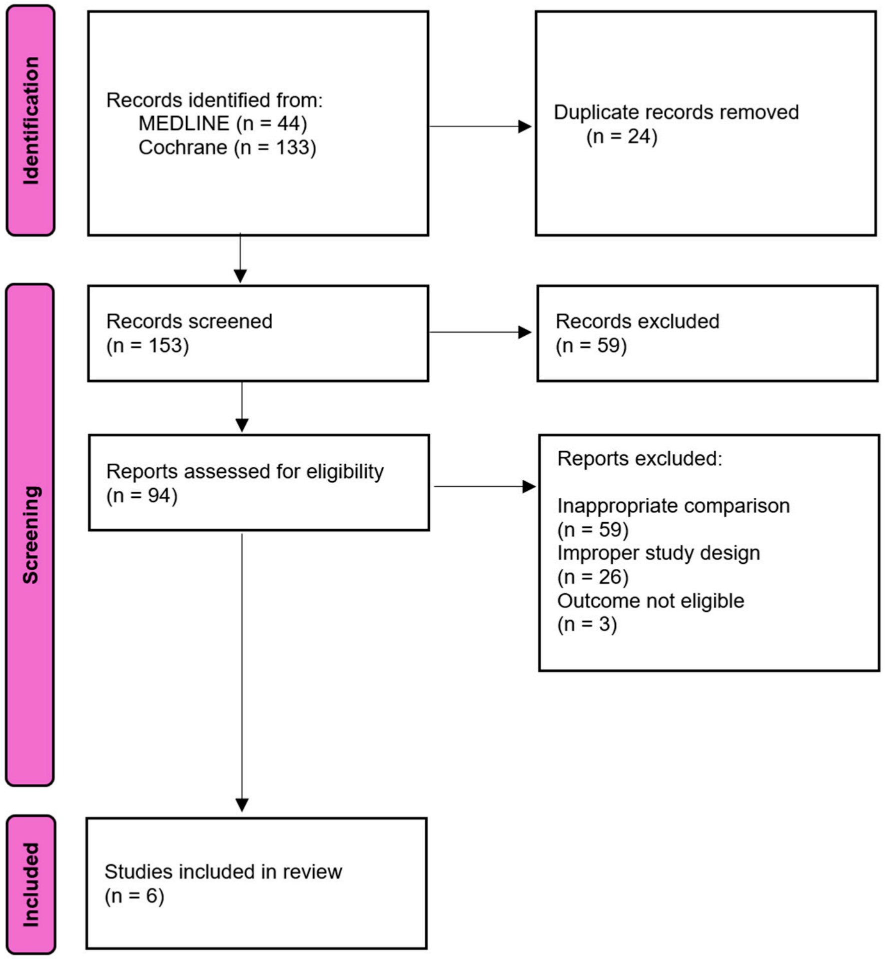

Figure S2. Risk-of-Bias Assessment

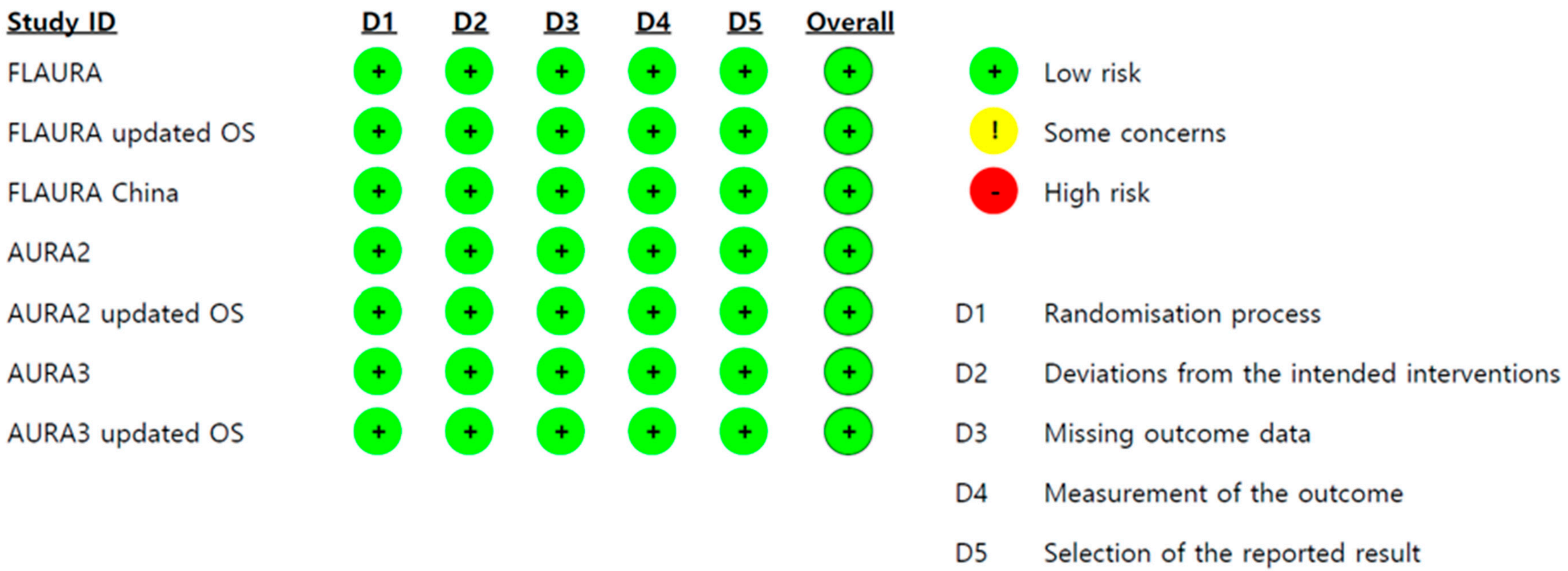

Figure S3. Comparisons of Original Curves and Reconstructed Curves in OS and PFS

Study, outcome, cohort	Original	Reconstructed
**FLAURA updated OS,**<br>**Overall survival,**<br>**Asian**	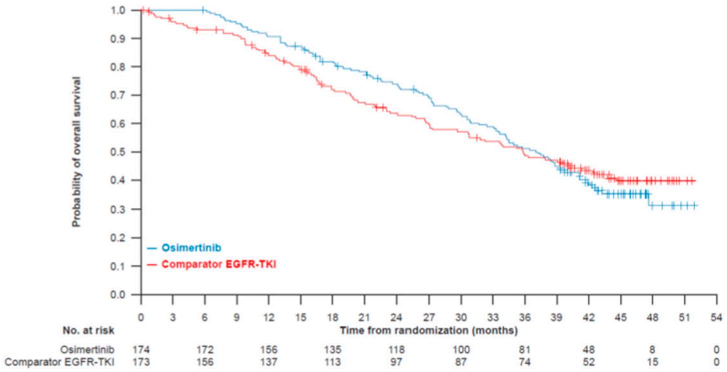 	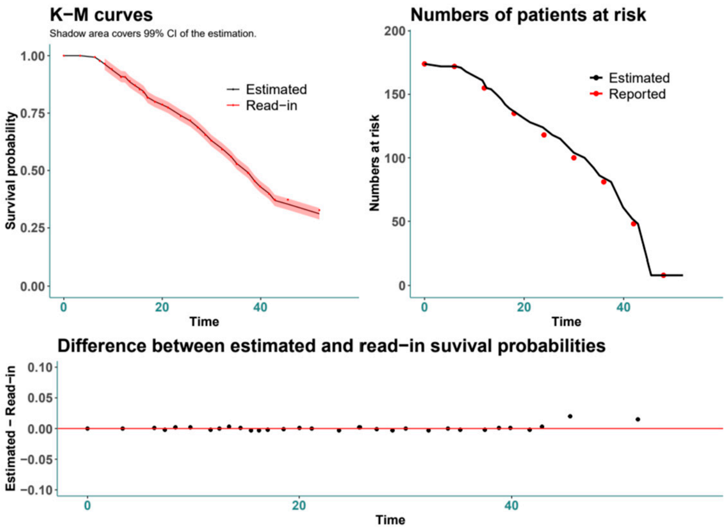

<table>
<tr>
<td>FLAURA updated OS,
Overall survival,
Non-Asian</td>
<td>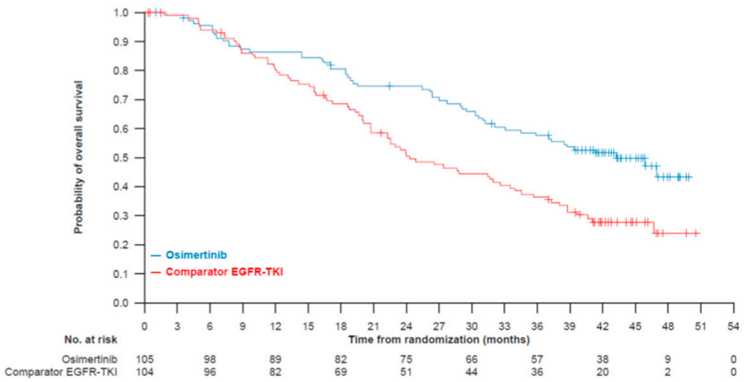
</td>
<td>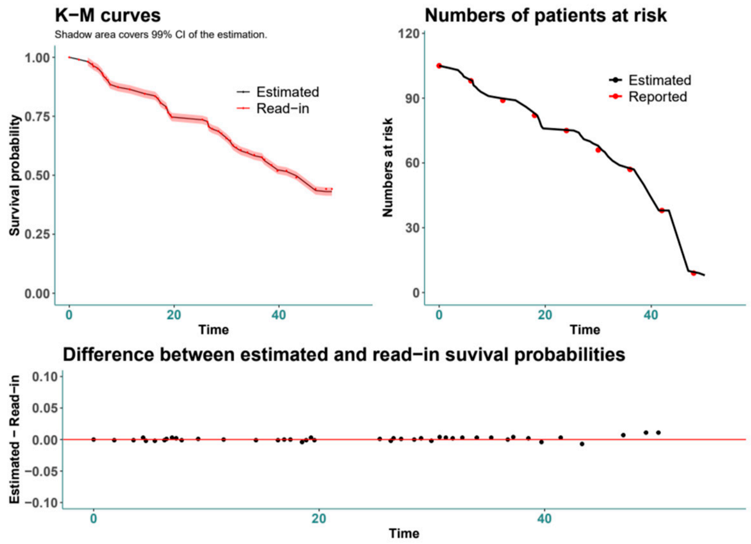
</td>
</tr>
</table>

**FLAURA,**<br>**Overall survival,**<br>**overall cohort**	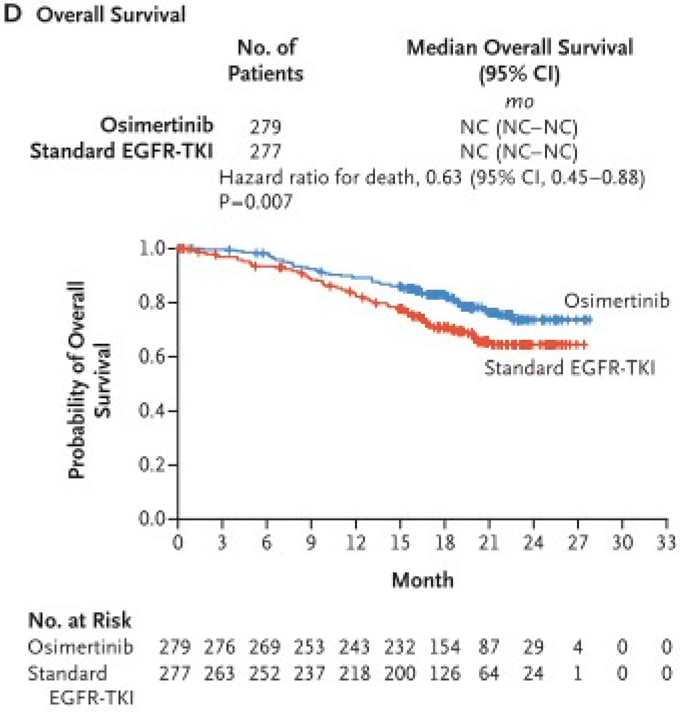	**NA (use updated OS data)**

FLAURA, Progression-free survival, overall cohort

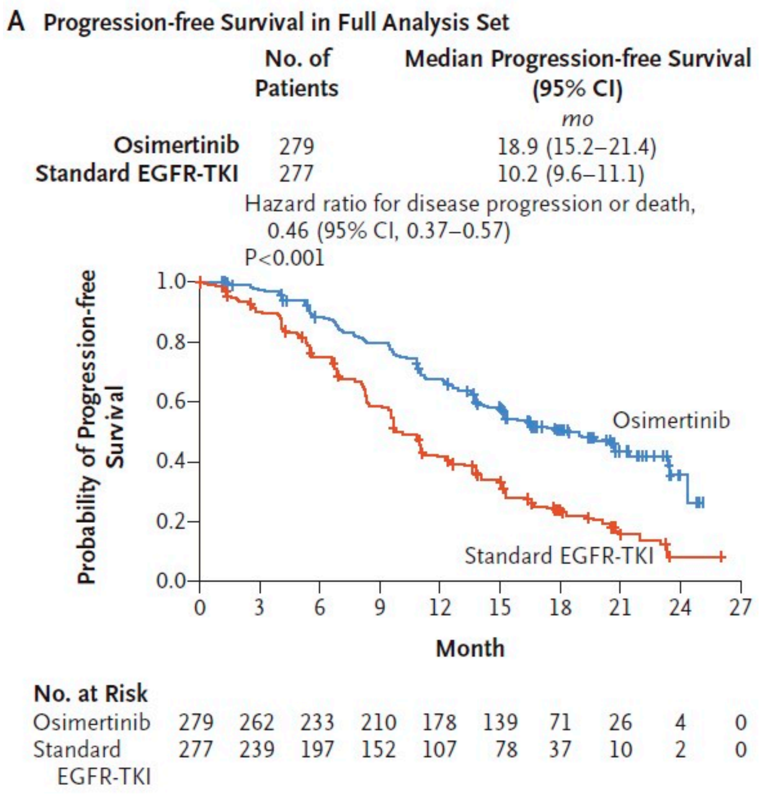

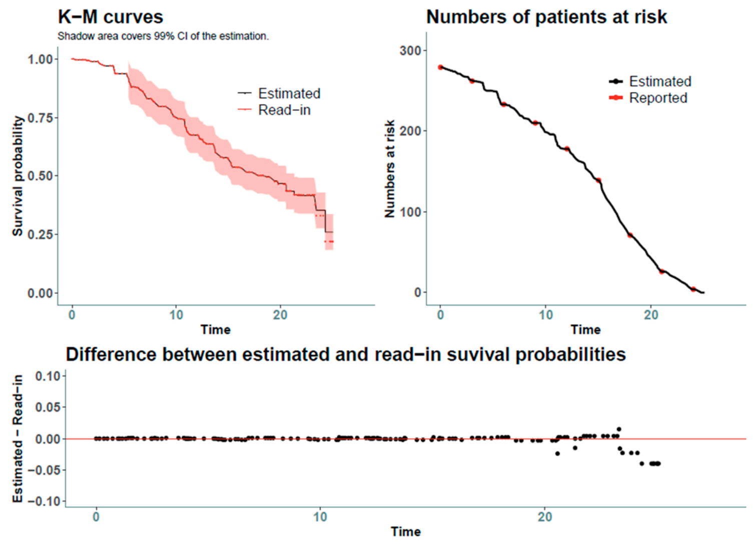

FLAURA China Overall survival, overall cohort

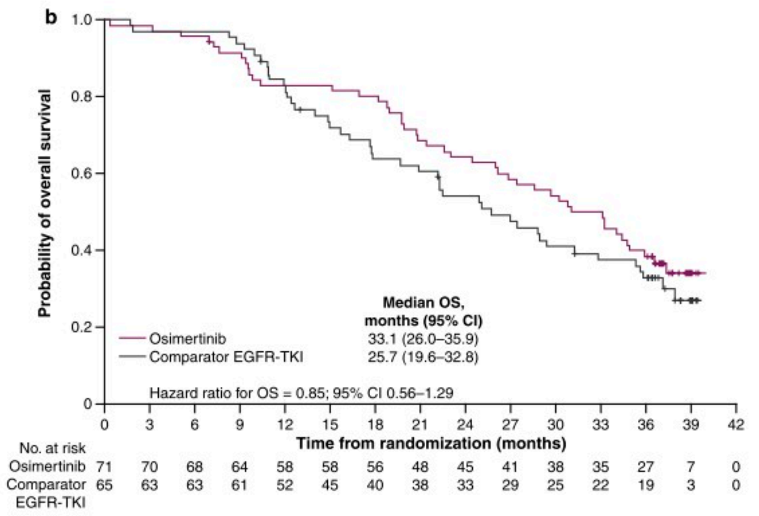

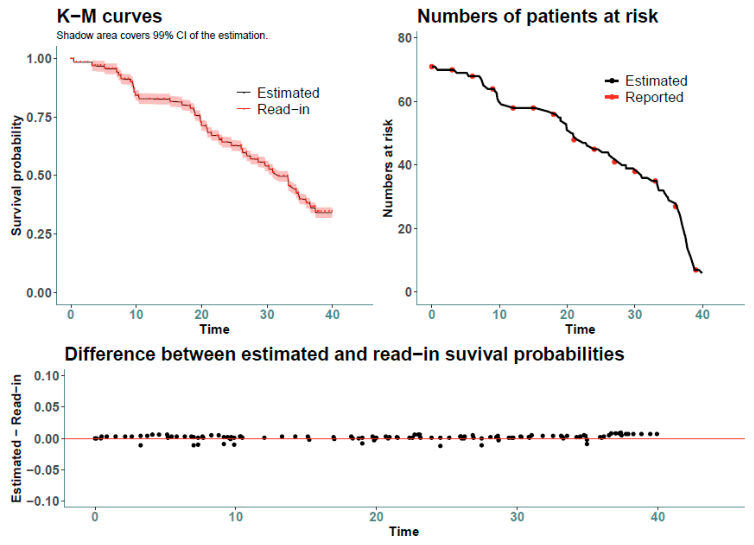

**FLAURA China Progression-free survival, overall cohort**	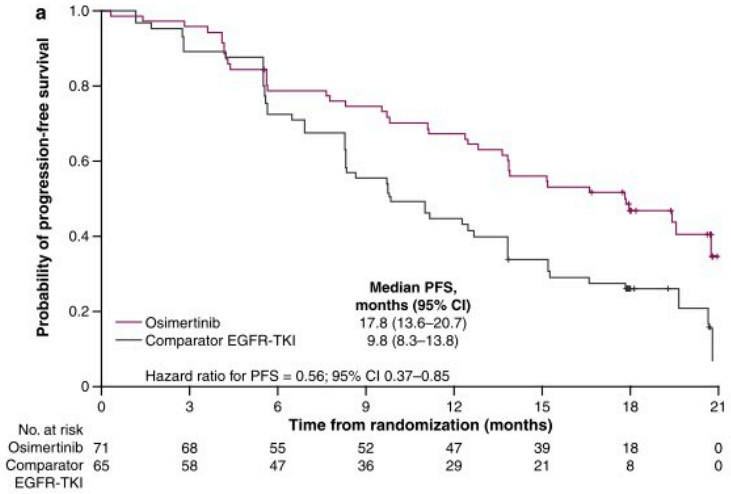	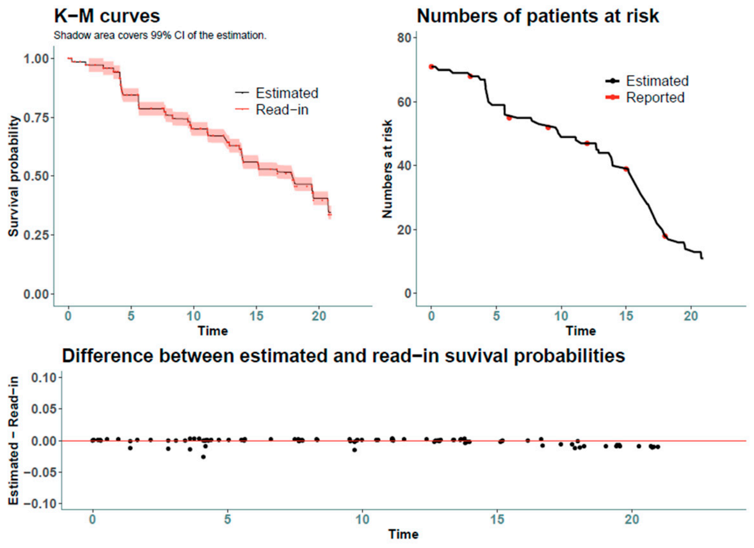

AURA2,

Overall survival,

Second-line cohort

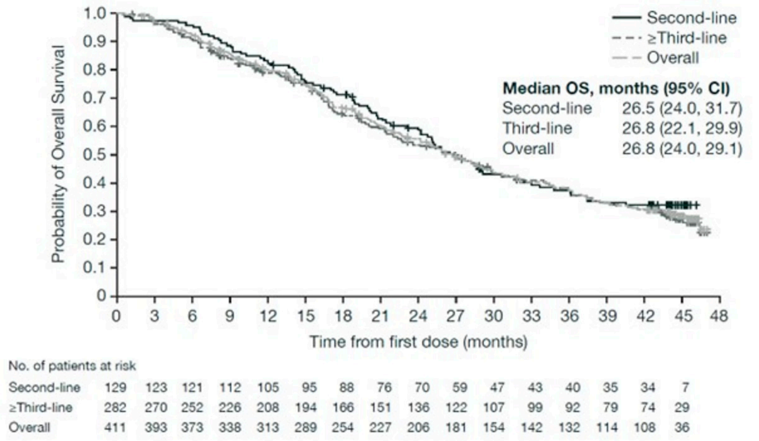

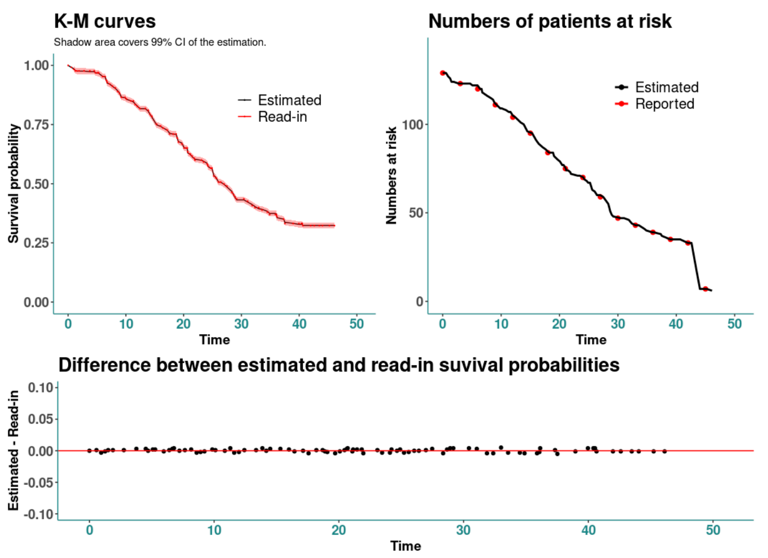

**AURA2, Progression-free survival, Second-line cohort**	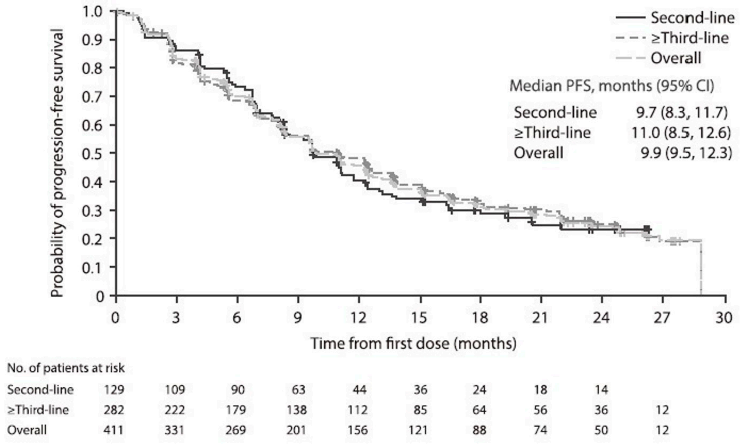	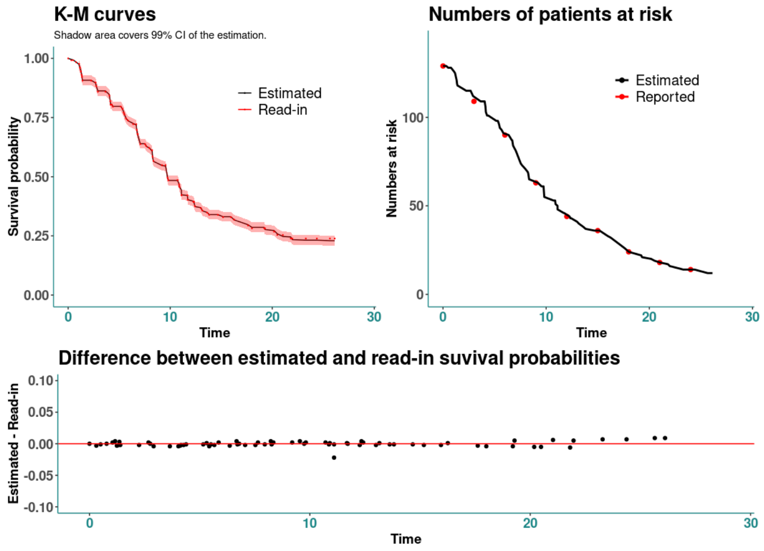

**AURA3 updated OS, Overall survival, overall cohort**	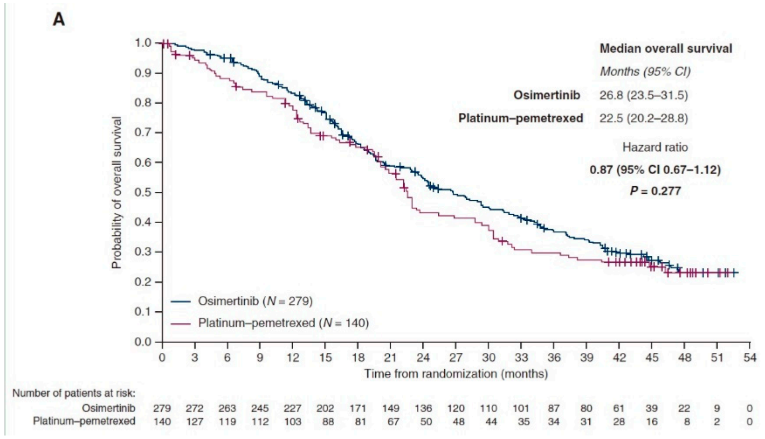	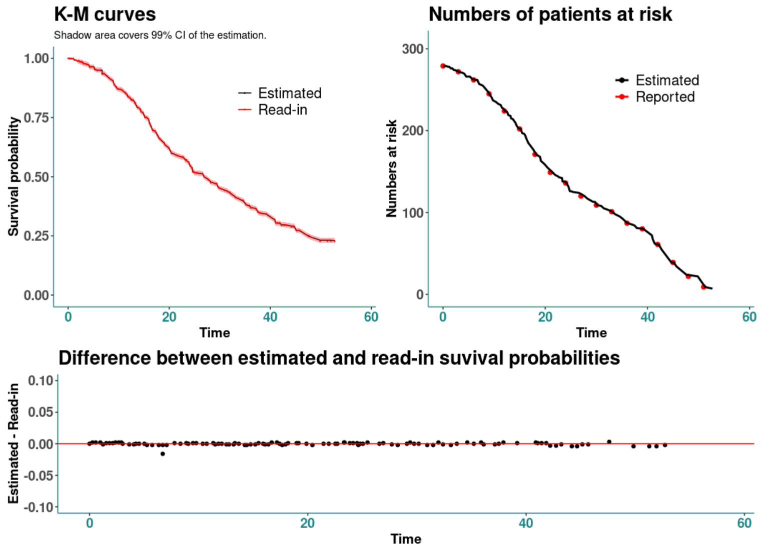
**AURA3, Overall survival, overall cohort**	**NA (use updated OS data)**	**NA (use updated OS data)**

<table>
<tr>
<td>AURA3,
Progression-free
survival,
overall cohort</td>
<td>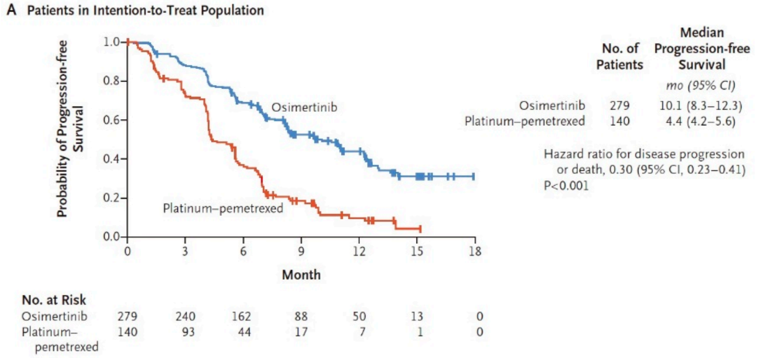
</td>
<td>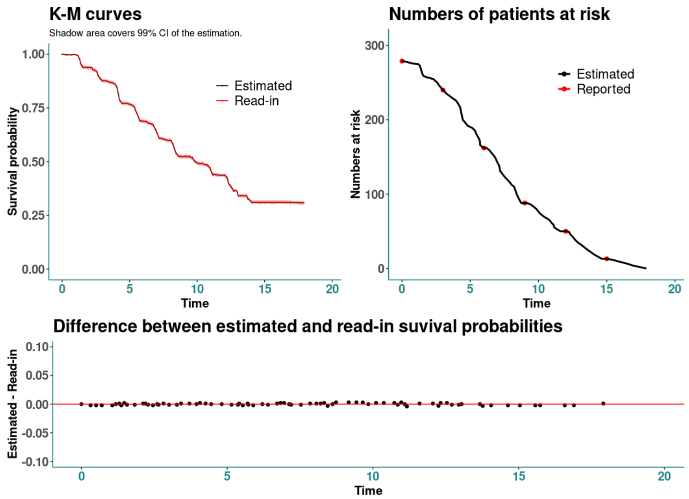
</td>
</tr>
</table>

Figure S4. Visual predictive check for the final OS models as the first-line treatment (A) and second-line treatment (B), respectively.

(A) VPC for the final OS models as the first-line treatment

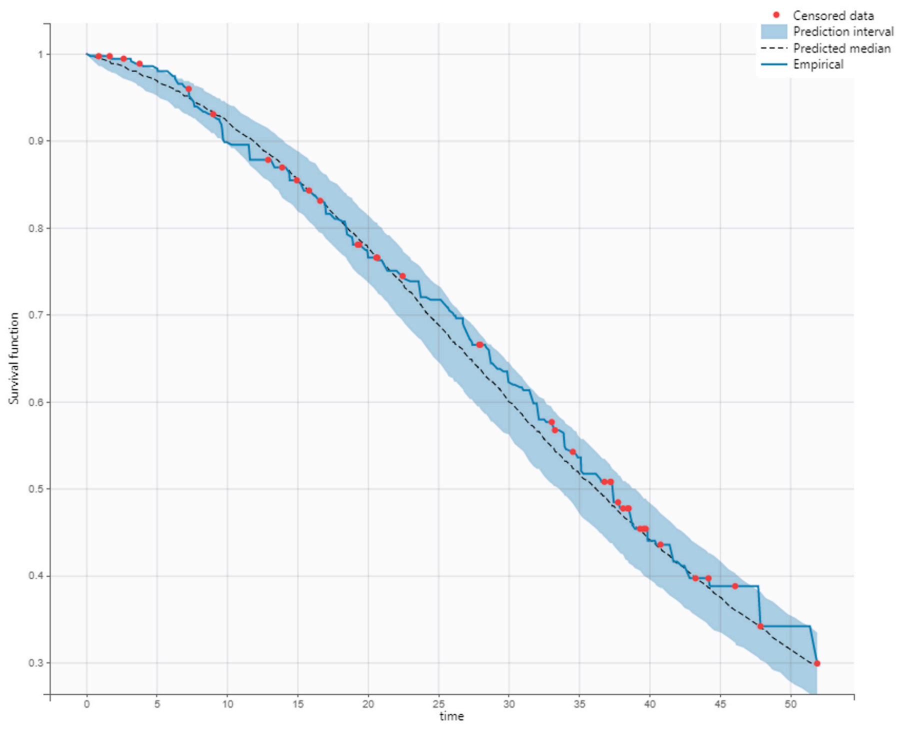

(B) VPC for the final OS models as the second-line treatment

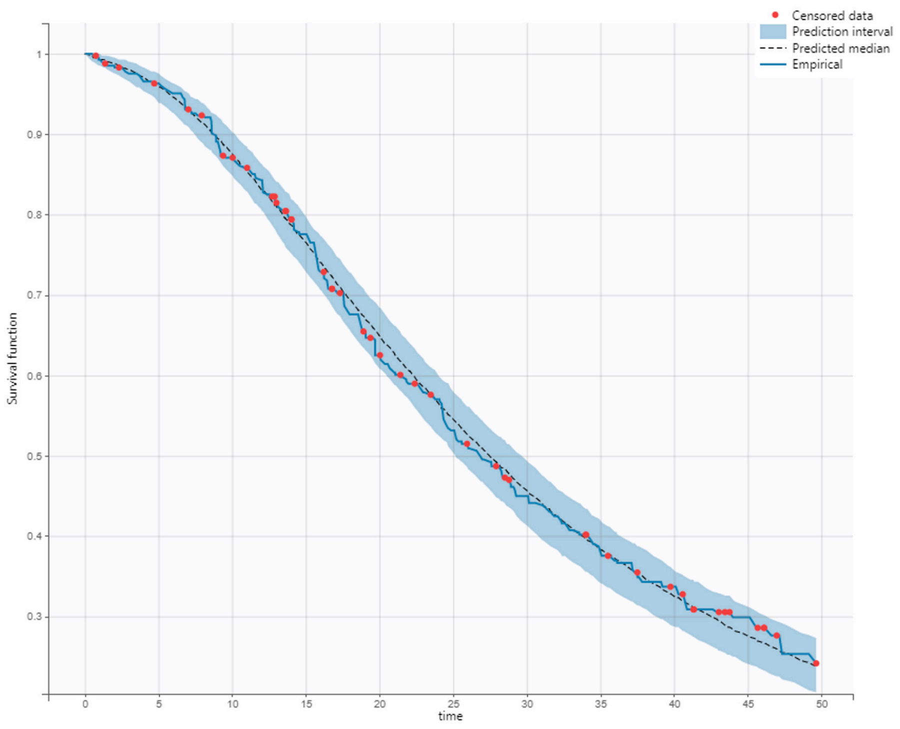

Figure S5. Visual predictive check for the final PFS models as the first-line treatment (A) and second-line treatment (B), respectively.

**(A) VPC for the final PFS models as the first-line treatment**	**(B) VPC for the final PFS models as the second-line treatment**
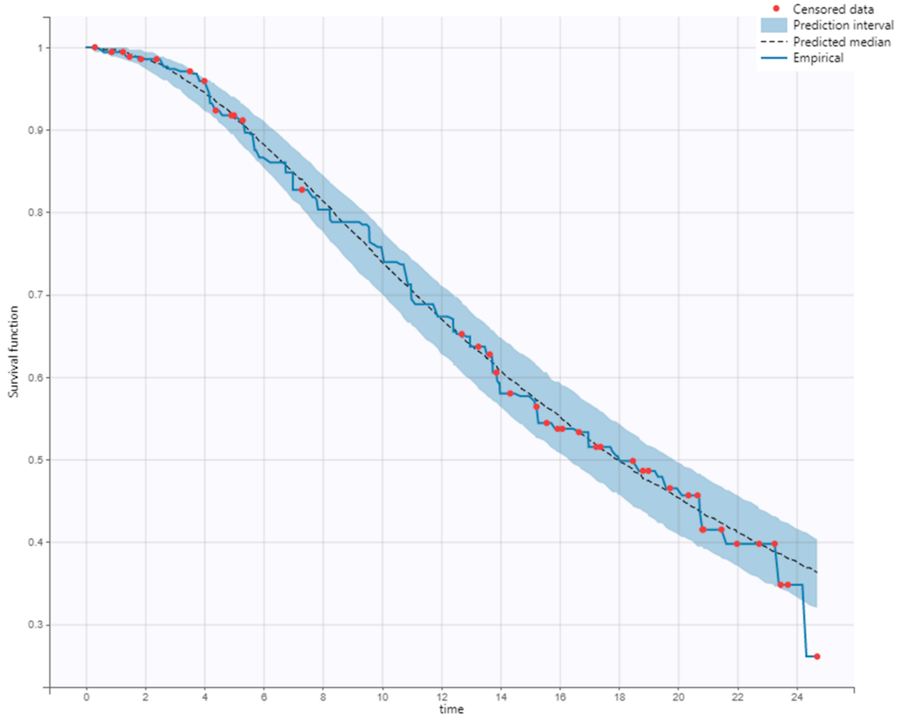	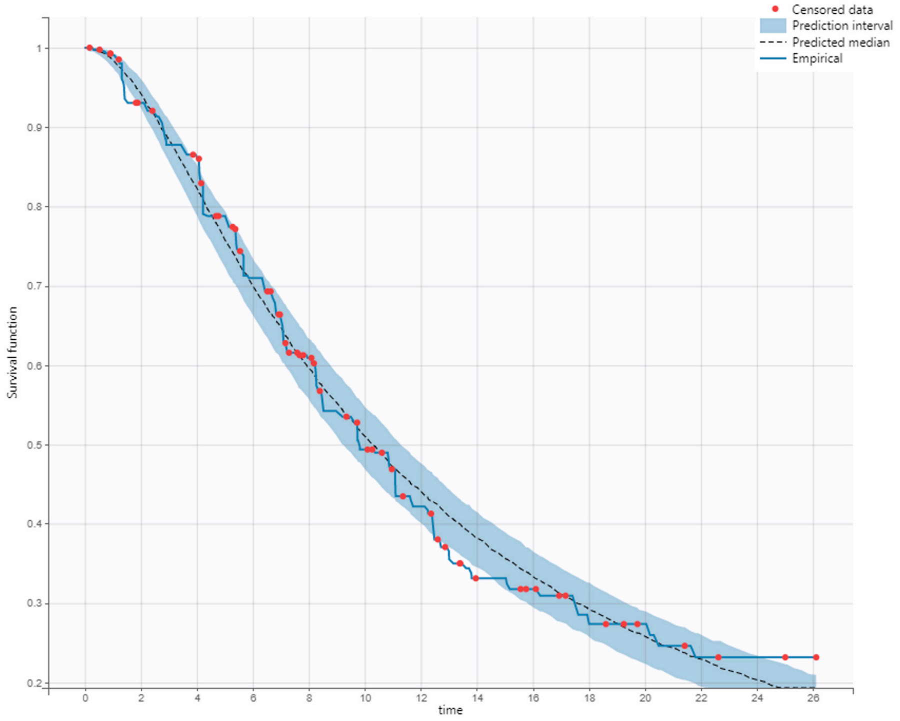
